# Time series outlier removal and imputing methods based on Colombian weather stations data

**DOI:** 10.1007/s11356-023-27176-x

**Published:** 2023-05-11

**Authors:** Jaime Parra-Plazas, Paulo Gaona-Garcia, Leonardo Plazas-Nossa

**Affiliations:** Universidad Distrital Francisco José de Caldas, Carrera 7 No. 40B-53 piso 9, Bogotá, Colombia

**Keywords:** Fast Fourier transform, Missing data imputing, Multivariate imputation by chained equations, Outlier removal, Time series, Weather station

## Abstract

The time data series of weather stations are a source of information for floods. The study of the previous wintertime series allows knowing the behavior of the variables and the result that will be applied to analysis and simulation models that feed variables such as flow and level of a study area. One of the most common problems is the acquisition and transmission of data from weather stations due to atypical values and lost data; this generates difficulties in the simulation process. Consequently, it is necessary to propose a numerical strategy to solve this problem. The data source for this study is a real database where these problems are presented with different variables of weather. This study is based on comparing three methods of time series analysis to evaluate a multivariable process offline. For the development of the study, we applied a method based on the discrete Fourier transform (DFT), and we contrasted it with methods such as the average and linear regression without uncertainty parameters to complete missing data. The proposed methodology entails statistical values, outlier detection, and the application of the DFT. The application of DFT allows the time series completion, based on its ability to manage various gap sizes and replace missing values. In sum, DFT led to low error percentages for all the time series (1% average). This percentage reflects what would have likely been the shape or pattern of the time series behavior in the absence of misleading outliers and missing data.

## Introduction

In engineering, time series are important because they show the behavior or pattern of one or more variables and their changes, and these time series are framed in the forecasting area. Basically, it has successive time events that can be characterized by amplitude values (Plitnick *et al*. 2018). Those values can have different interpretations, depending on the variable type as electrical, environmental, mechanical, biological, medical, thermal, and others. The time series study is important to understand how several variables interact with each other. In the changes in technology, especially in new applications through the Internet of things (IoT) as developed by Huang *et al.* (2017), this technology connects all devices located in different locations all the time, and they are constantly sending data that contains the variable values. This infrastructure sends and transmits a large amount of data, and it is stored in the database (DB), this implies the data analysis and pre-treatment using different algorithms to detect missing data and extreme values or outliers.

**Fig. 1:**
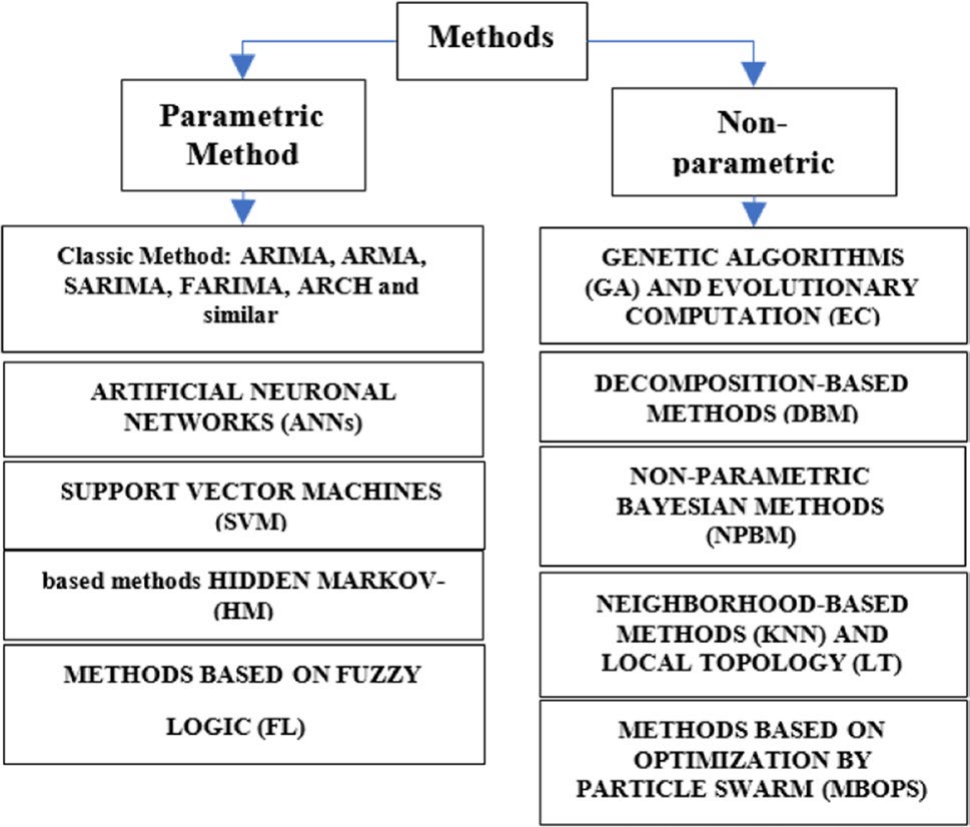
Classification of time series analysis methods

Time series can be evaluated in three different parts: (a) trend, which represents the main time series behavior and can be defined as non-strong changes compared to the main pattern; (b) seasonality, periodic changes that occur in regulars intervals; and (c) random, erratic changes that do not follow specific patterns, and they are due to different causes (i.e., device fails, wrong calibration processes, and environmental effects). This time series component has an unpredictable behavior, which is difficult to quantify and remove. As an example, the weather time series have variables that could include these components. These three parts or components represent the most types of changes in a time series (Rodríguez, 2016). The time series theory presents several numerical models to time series analysis, and they can be classified as shown in Fig. 1. The classification is divided into two aspects: the first is parametric methods and is composed of classical methods such as (a) ARIMA, ARMA, and SARIMA, among others; (b) ANN; (c) SVM; (d) HM; and (e) FL. The second is based on non-parametric, which are (a) GA and EC, (b) DBM, (c) NPBM, (d) KNN, and (e) MBOPS. These methods allow having a number of options for the study and analysis of time series, and for Schmitt et al. (2015) and Moritz et al. (2015), they study these methods and compare their results that show the possibilities of applications in projects where time series are carried out. With this variety of methods, a methodological development that is efficient in time processing is required to obtain adequate results for a high data volume analysis, see Fig. 1.

The above time series components are due to the physical phenomenon that produces it, beyond the measurement that can be attributed to the sensor reading and transformation processes, followed by transmitting it using telecommunication techniques whether wired or wireless. However, two problems are presented in time series as outlier values and missing values, and these two aspects have an important impact on the time series analysis process (Matlalcuatzi 2006). The above implies two processes: how to detect and remove the extreme values (outliers) and to establish the process to complete or impute the missing value. The outlier values must be removed because they are not within the time series value range, they cannot be detected in a simple way, their elimination must be careful, and this implies a previous analysis (Plazas-Nossa, 2016). The problem of outlier values in time series has been reviewed whether univariate or multivariable. Different methods have been approached for the development of an elimination criterion, but there is not a single model that allows the elimination of those extreme values. Montgomery et al. (2016) have compiled most of the usual methods developed for mathematical and numerical time series analysis. These methods are applied to atypical value estimation and how to eliminate them from the time series. Thus, some of the related univariate methods are mentioned as (a) regression methods, (b) autoregressive integrated moving average (ARIMA) smoothing methods, and (c) transfer functions and intervention models and some multivariate models as (a) multivariate time series models and forecasting, (b) state space models, (c) ARCH and GARCH models, (d) direct forecasting of percentiles, (e) combining forecasts to improve prediction performance, (f) aggregation and disaggregation of forecasts, (g) neural networks and forecasting, (h) spectral analysis, and (i) Bayesian methods in forecasting.

On the other hand, data loss is another aspect that has been studied in different areas. Several algorithms have been proposed to complete the lost data as (a) linear regressions (LR), (b) non-parametric regression (NPR), (c) principal component analysis (PCA), (d) imputation-based methods, and (e) neural network algorithms (NNA), among others. For Okoli et al. (2019), they developed a systematic and comparative review of statistical methods and created a model using synthetic data and assigning an uncertainty parameter and limiting the error, which allows estimating flooding, as well as involving flood risk reduction experts. The loss of data that is captured by the meteorological stations, and the transmission of the same has a probability of loss due to the deficiencies in the sensors and the connection and transmission of data, for this situation has to find a way to establish algorithms that allow to reconstruct the time series of data. For Khayati et al. (2020), they implemented an experiment with twelve algorithms and indices, and the result allowed to identify the limitations and that the algorithms do not offer a high accuracy and methodology of how to analyze the algorithms. Also, for Nor et al. (2020), a comparative model of imputation methods for daily rainfall data was implemented and based on linear and non-linear stochastic and index statistic methods to determine the performance of the algorithms and that the random forest–coupled method with multiple linear regression (RF-MLR) shows adequate performance for time series data loss imputation. And Afrifa-Yamoah et al. (2020) implemented a model based on a Kalman filter, an autoregressive integrated moving average (ARIMA), on twelve-monthly time series, on variables such as hourly temperature, humidity, and wind speed resulting in good indicators under indices such as root mean square error and symmetric mean absolute percentage error and recommended to apply it to other meteorological variables. Another aspect is multiple imputation proposed by Hamzah et al. (2021), whose methods are in multiple regression, classification and regression tree, K-nearest neighbors, and robust random regression, resulting in the robust random regression imputation (RRRI) algorithm coupled with multiple linear regression (MLR) having the best performance for imputing data to hydrological data sets. And finally Duarte et al. (2022) published on how to complete data from traditional non-automated stations using statistical algorithms and compared with the global precipitation measurement (GPM) mission. The result was similar with the statistical models, but those based on regressions did not meet the expectations, and to replace this part, satellite-based models are used, being a new option to complete temporary rain data series.

The methodology used this work to fill the gaps (missing values) in time series, combines statistical values from Tukey (1977) and Acuña and Rodriguez (2004), and also uses DFT (discrete Fourier transform) and IFFT (inverse fast Fourier transform) to complete the time series. The DFT allows the time series analysis that facilitates the switch from the time domain to the frequency domain. Thus, this technique converts a finite number of equally spaced samples (discrete points) into a number of coefficients of a finite combination of complex sinusoids (harmonics), ordered by their frequencies that have the same number of sample values (Proakis & Manolakis 2007). Doing so ensures that the frequency domain is comprised of the same number of sample values as the previous time domain (Proakis & Manolakis 2007).

Ozakil (2014) presents a comparison of methods to complete the temporal series of climatological datai using three methods, which are (a) nearest neighbor method, (b) inverse distance weighting method, and (c) linear regression method. Also, Campozano et al. (2014) developed a comparison of 17 methods of how to complete missing data in rainfall time series, where the author used deterministic algorithms, and it variables were evaluated, such as temperature and rainfall. The result obtained was compared under metrics such as mean error (ME), root-mean-square error (RMSE), and mean absolute error (MAE). Results show that the regressions had better temperature performance, but multiple regression differs in comparison with the low number of stations involved. However, the rainfall imputing methods showed good results, using multiple regression due to the number of stations involved.

The main objective of this work is to evaluate three numerical models to complete or impute missing data and remove the outlier values of weather time series obtained by sensors installed in seven weather stations. Also, it is possible to determine which is the best numerical method compared with each other. This document is developed as follows: the “Background” section presents the background, concepts, and equations used to develop this work. The “Data and methodology” section presents the study site, the variables applied in this work, and the methodology itself. The “Methodology proposed for time series analyses” section defines the methodology for eliminating atypical and imputing the incomplete or missing data. The “Result” and the “Analysis and discussion” sections show the results and the discussion of them, respectively. Finally, the “Conclusion” section presented the conclusions and future work.

## Background

The theoretical development of these models of the detection of atypical values and of completing the missing values has been followed by the improvement in computational processing in both hardware and software. Next, I relate some recent documents that expose the compilation of algorithms destined to solve this problem of the time series of weather. Another aspect is the large amount of data that can be acquired, processed, and transmitted by means of communication, which implies that strategies are required to optimize and process this large amount of data, the development of outliers, and missing data detection models. Finally, some prediction algorithms complete the process of analyzing time series for flood analysis.

### Outlier

**Fig. 2:**
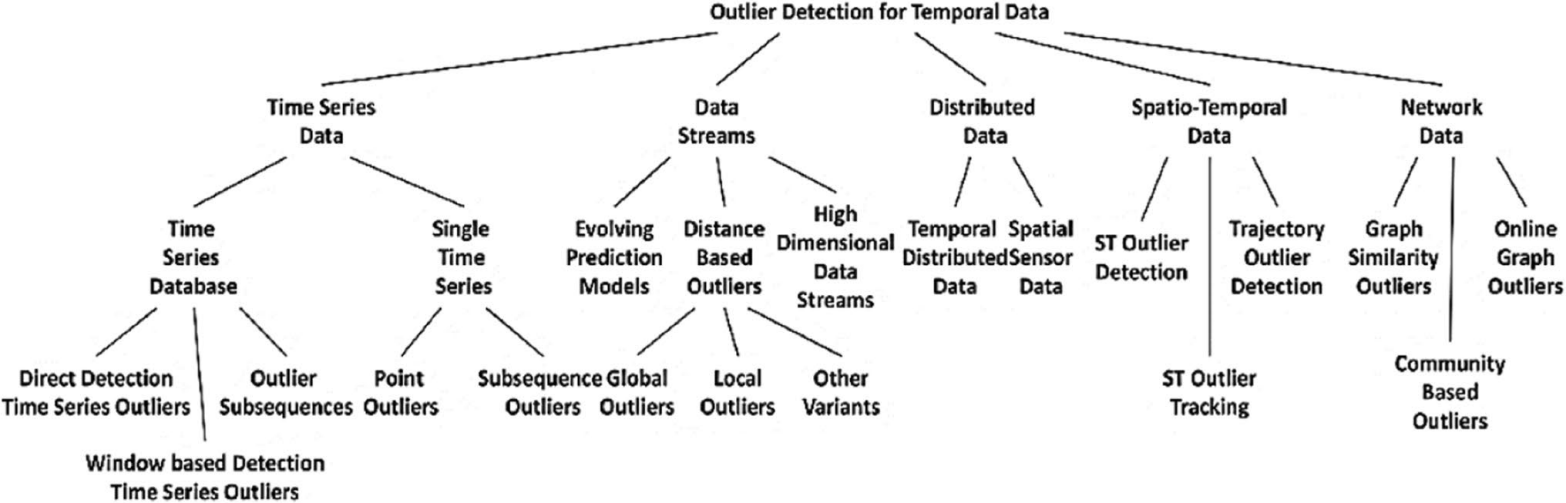
Classification of outlier values according to the source of the time series (Gupta *et al.* 2013)

An outlier is complicated to define clearly because this is not known in numerical value, but some authors have developed definitions that are generally enough and have been taken as a basis to define an outlier. Ben-gal’s study (2005) presents some of these: (a) an outlier as an observation that deviates from both other observations and to arouse the suspicion that it was generated by a different mechanism and (b) an outlier as an observation in a data set that appears to be inconsistent with the rest of that data set. Figure 2 presents the classification of atypical values according to the source of the time series of data, and this shows the diversity and the importance of how to detect them to eliminate them from the time series.

These outliers can be looked at from two aspects: (a) univariate methods and multivariate methods. (b) Another way to classify them would be of parametric (statistical) methods and non-parametric methods that are model-free. Since the review of literature in this issue of atypical values (Gupta *et al.* 2013) compiled extensive documentation of different areas of applications of time series and methods, among the conclusions found did not all solve the application of a generalized algorithm that allows to be applied to any data series. He also finds that it is required to work in areas that were abandoned as the methods based on Windows and finally presents a compilation of new techniques based on artificial intelligence algorithms, leaving a good field of action to continue in the investigation.

In the case of identifying atypical values of multiple data, Kannan and Manoj (2015) collect and test different methods such as Mahalanobis’ and Cook’s distance, leverage, masking effect, and diagnostics tool for statistical regression model (DFFITS) and as a result found that this methodology throws good results giving better performance the Cook’s distance method. Peng et al. (2016) treat the process of atypical detection by means of two generalized linear model (GLM) strategies and a proposed method defined by the generalized standardized corrected Pearson residuals (GSCPR). The first has problems due to the masking of atypical values and the second is the strategy of modifying the model based on the Pearson residual. The result is a notable improvement in the problem of masking and identifies the atypical values with the greatest number of successes, being a new strategy for multivariable series.

Another method of outlier detection is the arithmetic progression (AP) or arithmetic sequence that is a sequence of numbers with behavior: ascending, descending, or constant, so that the difference between the successive terms is constant, which can be described by Eq. 1.

1 $${a}_n=d\left(n-1\right)+{a}_1$$

where *d* is the difference or successive member and *a*_1_ is the first element of the series *n* which is *n*th term of the series. The development of the whole series is done with a sum of the finite elements AP with *n* elements and given by Eq. 2:

2 $${S}_n=\left(\frac{n}{2}\right)\ast \left({a}_1+{a}_n\right)$$

where *a*_1_ is the first element and *a*_*n*_ is the last element of the series. Then, we can consider that a straight line is a series without atypical values, and then, if there are atypical values, finite AP series does not comply with the straight line. This can be represented by Eq. 3.

3 $$\frac{2}{n}=\frac{\left({a}_1+{a}_n\right)}{S_n};\infty >n\ge 2,0<\frac{2}{n}\le 1$$

As a result, Eq. 3 is able to identify and locate atypical data, with a low percentage when they are close to real values. As a result of this numerical strategy, it is found that it is able to locate large and small atypical values.

To determine, if it is an outlier, a *Rw* criterion is established. Then, the value *Rw* is the factor that determines the value of the outlier if *w=0*, *R*_*0*_*=2/n*, and is a line and if *w > 0* is the edge of a line.

The value of *w* is $f\left(\frac{1}{n}\right)$. If the value *w* is $f\left(\frac{1}{n}\right)$ then $=2\ast \frac{k}{n}$, $k\le \left(\frac{n}{2}\right)-1$, and *k* ∈ *R*^+^. Then, $=\frac{2}{n+2}\ast \frac{k}{n}$ :

$${R}_w=\frac{2}{n}\ast \left(1+k\right)$$

4 $$\frac{R_w}{R_0}=1+k\ \textrm{where}=\textrm{cte}$$

The previous strategy in detail was developed by Adikaram et al. (2014). Being efficient in detecting outlier and eliminating it from the series. Then, to define the equations that evaluate whether the outlier is to the left or the right, Eq. 5 is defined as minimum, maximum, and sum of the series (MMS).


5


where *a*_max_ and *a*_min_ are the time series and *S*_*n*_ is the last of the series. *n* is *n*th term, and *w*_1_ is the factor that determines the outliers. In the case where the MMS does not detect the maximum or minimum of the series, then this is defined as a bad detection; in this case, MMS does not apply. An MMS improvement is then proposed and defined as an enhanced MMS series (EMMS) and is presented in Eq. 6 as


**Fig. 3:**
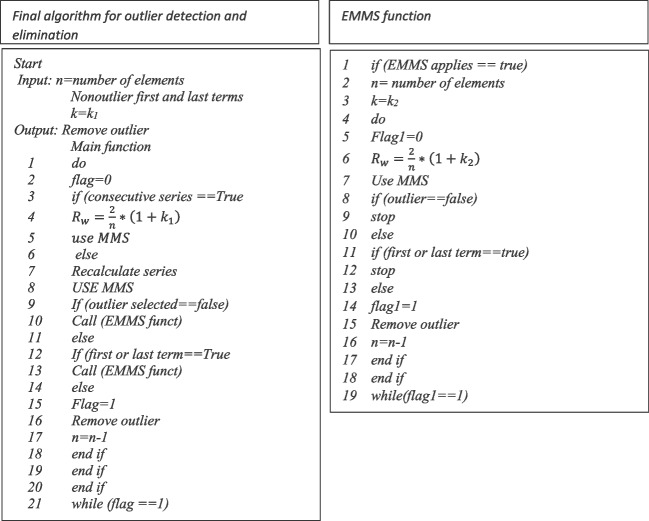
Outlier detection and elimination algorithm

6


where value transformation ${a}_{\textrm{max}}^{\textrm{TT}}$ and ${a}_{\textrm{min}}^{\textrm{TT}}$of the time series and ${S}_n^{\textrm{TT}}$ is the last of the series. *n* is *n*th, term and *w*_2_ is the factor that determines the outliers. Following, the outlier detection and elimination algorithm is described, which allows debugging the time series under study (Adikaram *et al*. 2014); this algorithm is able to identify atypical values that are significant and not significant, in addition to the fact that the algorithm is not parametric with floor and ceiling values. This implies that it does not require standardization. The periodicity of the data is wide, so any nonlinearity can be represented by a combination of straight lines. The pseudocode is shown in Fig. 3.

### Missing data

Establish a process to allow missing data due to factors such as data acquisition, transmission, and maintenance that is of vital importance. Campozano et al. (2014) present a compilation of 17 deterministic methods that allow filling those missing values in time series, and these methods are based on regression processes. The result obtained from this comparison shows that the multiple regressions with little information do not contribute to complete the missing information.

Another study, developed by Aslan et al. (2008), proposes an analysis of several methods to complete the missing data in the time series, the study was carried out on time series of meteorological stations, and the methods used are the simple arithmetic average (SAA), the normal relation (NR), NR weighted with correlations (NRWC), the multi-layer perceptron type of neural network (MLPNN), and the maximization of expectation algorithm based on the Monte Carlo Markov chain (EMMCMC). The result obtained shows that the EMMCMC method behaves very well before the deficiency of the lack of data in the series taken as a sample in the experimentation. It is necessary to have as much information as possible to have a better performance. Finally, in the study of Aissia et al. (2017), they developed a state of the art based on collecting different univariate and multivariable algorithms. As a result, it is necessary to improve the number of variables that can improve the results of the multivariate algorithms analyzed, in purchase with the algorithms of substitution and intermediate interpolation. Based on the above, an experimental model of elimination of atypical values and filling of missing data for a database is proposed, and the behavior of the various variables included in the database will be reviewed.

The detection and elimination of atypical data are done because the variables that are available from the weather station sensors are calibrated to measure an interval of possible data that is historically repeated in the time series and that has been collected for years. It is therefore that logical ranges are established for those variables and region that are the object of study by the Institute of Hydrology, Meteorology and Environmental Studies (IDEAM). Consequently, the data that is outside of this calibration is atypical, and for this reason, the elimination algorithm is implemented, which cleans the data to make way to complete the missing data and continue with the prediction process, either through model statistics or computational intelligence.

### Complete missing values of data

The sensor’s measurement process could be exposed to lost values, and these lost values might be owing to obstructions, clogging, failure, and/or maintenance periods. Thus, it is necessary to carry out a filling process applied to univariate and multivariate time series, which have missing values. The complete missing value procedure is part of the pre-treatment stage. Once the corrected time series (i.e., without outliers) is obtained, a DFT (discrete Fourier transform) procedure to complete the time series can be applied. The DFT (discrete Fourier transform) allows the time series analysis that facilitates the switch from the time domain to the frequency domain. Thus, this technique converts a finite number of equally spaced samples (discrete points) into a number of coefficients of a finite combination of complex sinusoids (harmonics), ordered by their frequencies that have the same number of sample values (Proakis & Manolakis 2007). Doing so ensures that the frequency domain is comprised of the same number of sample values as the previous time domain (Proakis & Manolakis 2007). This technique involves the conversion of a finite number of equally spaced samples (discrete points) into a number of coefficients that stem from a finite combination of complex sinusoid components. Doing so ensures that the frequency domain has the same number of sample values as the previous time domain (Proakis & Manolakis 2007).

Equation 8 details this conversion. DFT ranks harmonics based on their importance determined by harmonic amplitude. After ranking, harmonics are eliminated from lower to higher importance, leaving only the most significant harmonics in the resulting values. Finally, the inverse fast Fourier transform (IFFT) is utilized to convert complex sinusoids (harmonics) into a finite number of discrete points, returning to the time domain, as shown in Eqs. 7 and 8.

7 $${X}_k={\sum}_{n=0}^{N-1}{x}_n{e}^{-j2\pi \frac{k}{N}n}\kern1em k=0,1,\dots, N-1$$

8 $${X}_n=\frac{1}{N}{\sum}_{k=0}^{N-1}{X}_k{e}^{-j2\pi \frac{k}{N}n}\kern1em n=0,1,\dots, N-1$$

With DFT, the time series is transformed from the time domain to the frequency domain (Plazas-Nossa and Torres, 2013). Thus, the DFT is applied with the data set prior to the first missing values gap, and the ten (10) most important harmonics are selected in order to reproduce the pattern and dynamic of the time series. By IFFT, the data are converted from the frequency domain back into the time domain. The resulting time series is then used to complete the first gap of missing values. Therefore, the process described up to this point is brought to bear on the corrected data ranging from the starting point (including the first “completed” gap) to the beginning of the second gap. The same is done with all the corrected data from the initial point to the start of the third gap (including the “completed” first and second gaps) and so on; this is called the “forward step to fill (FSF).” However, if the half length of the available values range is lower than the length of the missing values gap, the procedure tests if the subsequent half length of the available value range is lower than the length of the gap. If it is lower, the values from that available range are used to fill the gap. Therefore, this step is called the “backward step to fill (BSF).” But if neither steps of the forward and backward filling have enough length, the total values, including available values and replaced values in previous ranges, from the start of the time series are used to fill the missing values gap; this is called “total amount of values step to fill (TSF)” (Plazas-Nossa et al., 2015).

**Fig. 4:**
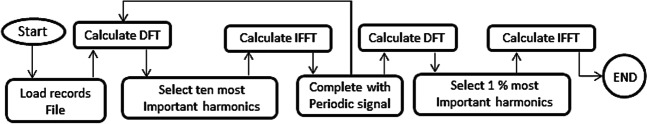
Flow diagram depicting replacement of missing values

Finally, DFT is applied to the resulting time series, and 1% of the most important harmonics is used. Going from the frequency domain back to the time domain is once again done with the IFFT process. At this stage, new values replacing either outliers or missing values have the same (or almost the same) shape as the original time series, granting the “macro” vision of the time series coherence. The flow diagram in Fig. 4 summarizes the complete process.

Other methods of detection of data loss and outliers are the following proposed model multivariate imputation by chained equations (MICE) exposed by Buuren and Groothuis-Oudshoorn (2011). Yakel (2004) and Yucel (2008) develop broadly the use of various techniques aimed at completing data and outlier detection. The R library called MICE is used, and the mean and norm.nob strategies are applied. The average function is described by means of Eq. 9:

9 $${p}_x=\frac{1}{n}\sum_{i=1}^n{p}_i$$

This allows to take the same weight for all the climatological stations, in the different variables to analyze (Caldera *et al.* 2016). The function norm.nob is based on the imputation by linear regression without parameter uncertainty algorithm, described for Eqs. 9 and 10.

10 $${\displaystyle \begin{array}{c}P\left({Y}_1|{Y}_{-1},{\theta}_1\right)\\ {}\vdots \\ {}P\left({Y}_p|{Y}_{-p},{\theta}_p\right)\\ {}{\theta}_1^{\ast (t)}\sim P\left({\theta}_1|{Y}_1^{obs},{Y}_2^{\left(t-1\right)},\dots ..{Y}_p^{t-1}\right)\end{array}}$$

11 $${\displaystyle \begin{array}{c}{Y}_1^{\ast (t)}\sim P\left({Y}_1|{Y}_1^{obs},{Y}_2^{\left(t-1\right)},\dots {Y}_{p-1}^{\left(t-1\right)},{\theta}_1^{\ast (t)}\right)\\ {}\vdots \\ {}{\theta}_p^{\ast (t)}\sim P\left({\theta}_p|{Y}_p^{obs},{Y}_1^{(t)},\dots .{Y}_{p-1}^{(t)}\right)\\ {}{Y}_p^{\ast (t)}\sim P\left({Y}_p|{Y}_p^{obs},{Y}_1^{(t)},\dots {Y}_p^{(t)},{\theta}_1^{\ast (t)}\right)\end{array}}$$

where ${Y}_j^{\ast (t)}=\left({Y}_j^{obs},{Y}_j^{\ast (t)}\right)$ is the *j*th imputed variable at iteration *t*. Observe that previous imputations ${Y}_j^{\ast \left(t-1\right)}$ and only enter ${Y}_j^{\ast (t)}$ through its relation with other variables, and not directly. These three methods will be used to establish which best performance has to be applied to outlier detection and data loss in transmission variables weather stations.

## Data and methodology

Below is a description of the data and the methodology to be developed.

### Study region

For the development of this proposal, we have a database of an area that was affected by floods with recurrence, the territory of the city of Ciénaga, Colombia; these data were provided by a network of stations with several meteorological stations with different sensors that monitor the area under study. Ciénaga is a town located in Colombia, and its topography is a swamp that flows into the sea. The topography is of a low terrain that has an average elevation above sea level of 10 m and an area of 1242.68 km^2^.

**Fig. 5:**
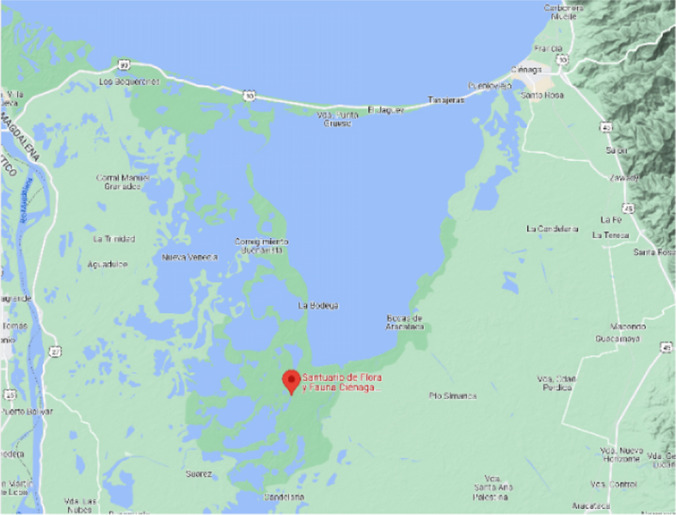
Geographical location Ciénaga Magdalena, Colombia (Google,2022)

Eighteen months of data were collected from 2016–2017 and a data transmission rate every 1 min with 10 weather stations distributed in the study area, see Fig. 4. The records were stored and reviewed for their atypical behavior and data loss. Then, we developed an experiment to establish which model is the most appropriate to complete the missing data and eliminate atypical data for this by using other data from nearby stations to achieve homogeneity of the data. The study area is a populated region that is affected in the winter seasons in the past, see Fig. 5.

With the above, the best model was established for the numerical analysis of atypical data and the loss of data to generate an efficient method to complete the information that is transmitted from the weather station through the cellular network in the area to a base of data on the Internet.

### Database and software

The database was developed in MySQL and has the following variables, which are presented in Table 1.

**Table 1:** List of database variables

Variable	Description
th0temp	Outside temperature in degrees Celsius
th0hum	Outdoor relative humidity as a percentage
Thdedew	Dew point outdoors in degrees Celsius
thb0press	Pressure in hPa
thb0seapress	Normalized pressure (calculated at sea level) in hPa
wind0wind	Wind speed in m/s
wind0dir	Average wind speed in m/s
wind0chill	Wind chill temperature in degrees Celsius
rain0rate	Rainfall index in mm/h
rain0total	Rainfall in mm
uv0index	Index ultraviolet
sol0rad	Solar radiation in W/m^2^
sol0evo	Evapotranspiration in mm (only compatible with Davis Vantage stations)
sun0total	Duration of sunlight in hours (only compatible with Davis Vantages equipped with a third-party sunlight detector)

The database was built on a platform for transmission of data through the Internet, and 10 weather stations were installed in the area of influence of Ciénaga Magdalena; each station transmitted data of 14 environmental variables, and finally, 8 meteorological stations are taken for having consecutive data and 9 variables for the experiment. The information collected has approximately 18 months of records between the years 2-08-2016 and 08-25-2017. The total number of records is approximately 3,827,450, which must be reviewed to determine outliers and lack of information. Table 2 is a summary of the 8 stations taken for the study and the amount of data for each one. Table 2 shows the amount of data recorded by each of the weather stations.

**Table 2:** Summary of the 8 stations and data

Variable:idestadistics	Name station	# Register
E1	Kenedy	438042
E2	San Pedro	563359
E3	Cordobita	469851
**E4**	**Palmor**	**549328**
E5	La Bodega	537145
E6	El Cenizo	544574
E7	Sevillano	539413
E8	Siberia	185738

Part of the experimentation is to know the behavior of the time series of data provided by these stations and to evaluate two aspects: atypical and lost data in a multivariable model where the objective is to establish what proportion of atypical data and missing data is found in the time series. As a result, develop a numerical strategy that completes the data and obtains homogeneity in the data for the next prediction phase. The software used for the processing is R studio version 1.1.447 © 2009–2018 R studio, Inc., tool of R that provides a great amount of functions for the processing of the data.

## Methodology

The methodology used was based on the following stages: first, establish the amount of information recorded DB and the behavior of the time series according to Jain and Kumar (2007) and Mudelsee (2010) of each of the available variables. It was defined which variables are applied to the experimental process, since not all of them are necessary for the precipitation forecast and the stations that apply of the eight available according to the data of Table 2.

The behavior of the data was taken into account, and the development of a numerical model that detects atypical and missing data is considered. For this activity, Attah’s model (2011) is used, which explains the kinetic and dynamic types, followed by defining which models can be tested to be applied in the cleaning of the time series of data. This allowed us to review if it is necessary to treat the data through univariate or multivariable methods.

With respect to the lack of data and outliers, the objective was to evaluate what percentage of the total data. From the review of the state-of-the-art techniques, it is important to use and have at least several methods both in the process of atypical data and in the loss of data, centered on the deterministic, stochastic, and artificial intelligence methods for this part, from Chawsheen and Broom (2017), Rahman and Lateh (2017), and Robichaud and Comtois (2017). As a methodological model, we use the strategy (Yang *et al*. 2017), which describes in detail each phase to eliminate the outlier and complete the lost data, in our case using the AP method, followed by the mean, stochastic regression, and DFT methods for complete data. As part of the discussion to propose the methodological model, Aguasca-Colomo et al. (2019) and Papailiou et al. (2022) presented the treatment of data from meteorological stations in terms of the pre-processing of temporary data series, seeking to determine the behavior of the loss of data. For the methodology of eliminating the outlier data, Kulanuwat et al.’s (2021), Azman et al.’s (2021), and Baddoo et al.’s (2021) study were reviewed with the aim of establishing a methodology that allows verification with statistical tests to determine the error and likewise establish the best method. And finally, the methodology of Chiu et al. (2021) and Addi et al. (2022) for the analysis of results under statistical models such as root mean square error and mean absolute error (Duarte *et al*. 2022) presents similar statistical indicators. Finally, it was evaluated comparatively; therefore, the best behavior among the three methods was defined for its final implementation and thus completing the database for the next phase of the forecast project under a standard.

## Methodology proposed for time series analyses

**Fig. 6:**
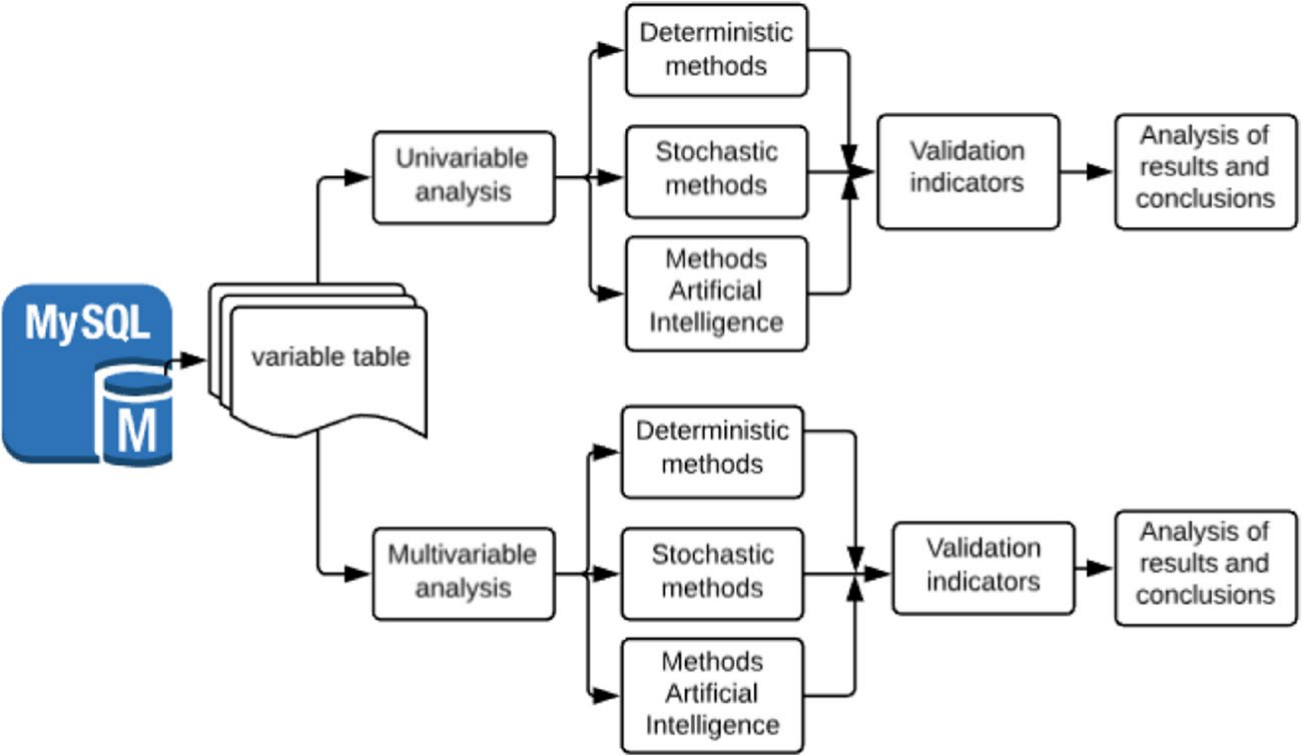
Classification model for the analysis of time series

The model of the present proposal has two aspects for the analysis of time series: univariate and multivariate because the available database has enough information for the development of both options. The classification of these methods is shown in Fig. 6.

**Fig. 7:**
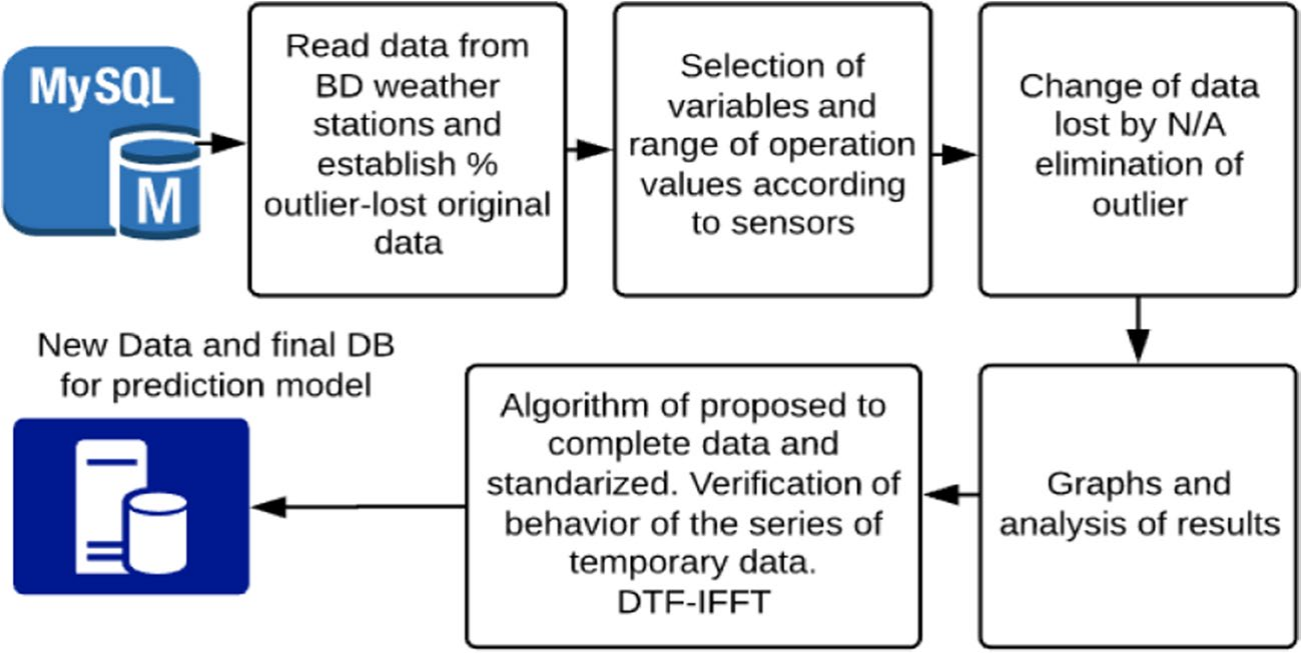
Process of identification and elimination of outlier and loss of data

According to Fig. 6, the implementation of the model of completing the data based on the numerical strategy of Fig. 3 is proposed and is integrated into the proposal in Fig. 6. The strategy of completing data is done using DTF-IFFT. Apply to one of the 10 meteorological stations identified as E4, and take the ten variables from Table 1. Based on this classification we propose a model of outlier detection and data loss, thus see Figure 7:

It is necessary to use a strategy comparison between the original set of time and the result of the DFT-IFFT process using index normalized quadratic mean deviation (NRMSD) expressed in Eq. 12.

12 $$\textrm{NRMSD}=\frac{\sqrt{\frac{\sum_{i=1}^n{\left({\textrm{Val}}_{{\textrm{IFFT}}_i}-{\textrm{Val}}_{{\textrm{ST}}_i}\right)}^2}{n}}}{{\textrm{Obs}}_{\textrm{max}}-{\textrm{Obs}}_{\textrm{min}}}\ast 100$$

where ${\textrm{Val}}_{{\textrm{ST}}_i}$ is the original time series value for time index *i,* ${\textrm{Val}}_{{\textrm{IFFT}}_{i,}}$ is the variable value after the deletion process for time index *i*, Obs_max_ − Obs_min_ is the original time series amplitude range, and *n* is the number of the forecasting value. Another aspect, the data sets collected by the sensors of the meteorological stations are not within the values of the range of the sensor, due to maintenance processes, energy, or hardware variations that require the restart of the electronic system. For these conditions, a filter is applied to the data sets by means of the gradient operator, applying linear interpolations in order to complete the time series, Eq. 13.

13 $$\left|\frac{{\textrm{Value}}_i-{\textrm{Value}}_{i-1}}{\textrm{Delta}}\right|\le 1$$

where is the Value_*i*_ is the value variable for time *t*, Value_*i* − 1_ is the value for time *i-1*, and Delta is the range of the variable chosen arbitrarily. Based on this methodology, the following results are applied to the station defined as E4 (Table 2) that has the largest amount of data and the best coherence in the transmission of the data, being the test algorithm for final implementation within the research project “Model of Prediction of Flooding Through the Use of Computational Intelligence Techniques.”

## Result

**Fig. 8:**
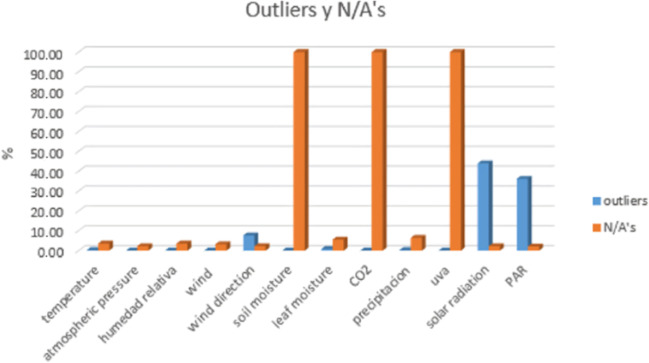
Percent result % of outlier and original lost data of stations

In the first place, the percentage of outlier and missing data is evaluated from the DB and thus reduces the number of variables to be studied from stations this is presented in Fig. 8.

As can be seen in Fig. 7, the variable soil moisture, CO_2_, and ultraviolet radiation (UVA) have a large percentage of loss and are excluded from the process because there is not enough information to develop the process of elimination and complete its high percentage of few data. Within the evaluation of the 10 weather stations, the one that has the largest amount of information and that presents a percentage of lost data that helps generate the data that is needed is selected. The ranges of each of the meteorological variables are established, and Table 3 of station E4 is presented, where the atypical values have been eliminated, leaving only the N/A part and ready to develop the three filling models.

**Table 3:** Summary of the 8 stations

Variable	Unit	min	max	N° Reg N/A	Total Reg
Temperature	°C	15.5	30.4	355855	1048575
Atmospheric pressure	Mbar	898	909	355854	1048575
Relative humidity	% HR	41.1	100	355885	1048575
Wind speed	m/s	0	12.1	355856	1048575
Direction of the wind	DEG	0	76	733879	1048575
Leaf moisture	%	4.1	100	355854	1048575
Precipitation	mm H_2_O	0.00	2.8	356089	1048575
Solar radiation	W/m^2^	1	1279	355853	1048575
Par	μmoles photon	1	2559	355909	1048575

**Fig. 9:**
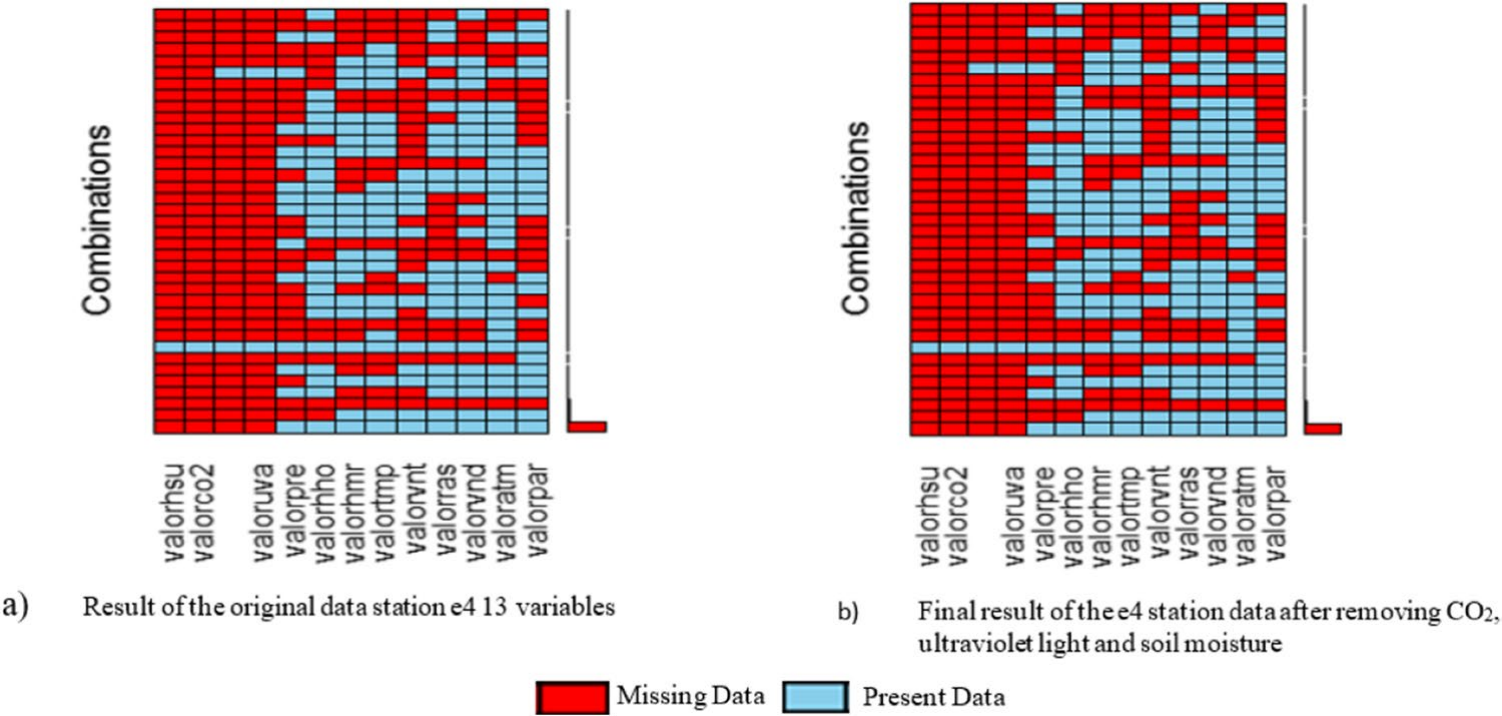
**a**, **b** Result of the elimination of three variables and outlier station E4

Figure 9 shows how the variables remain before and after eliminating 3 variables and outlier for the process of completing the missing data.

**Fig. 10:**
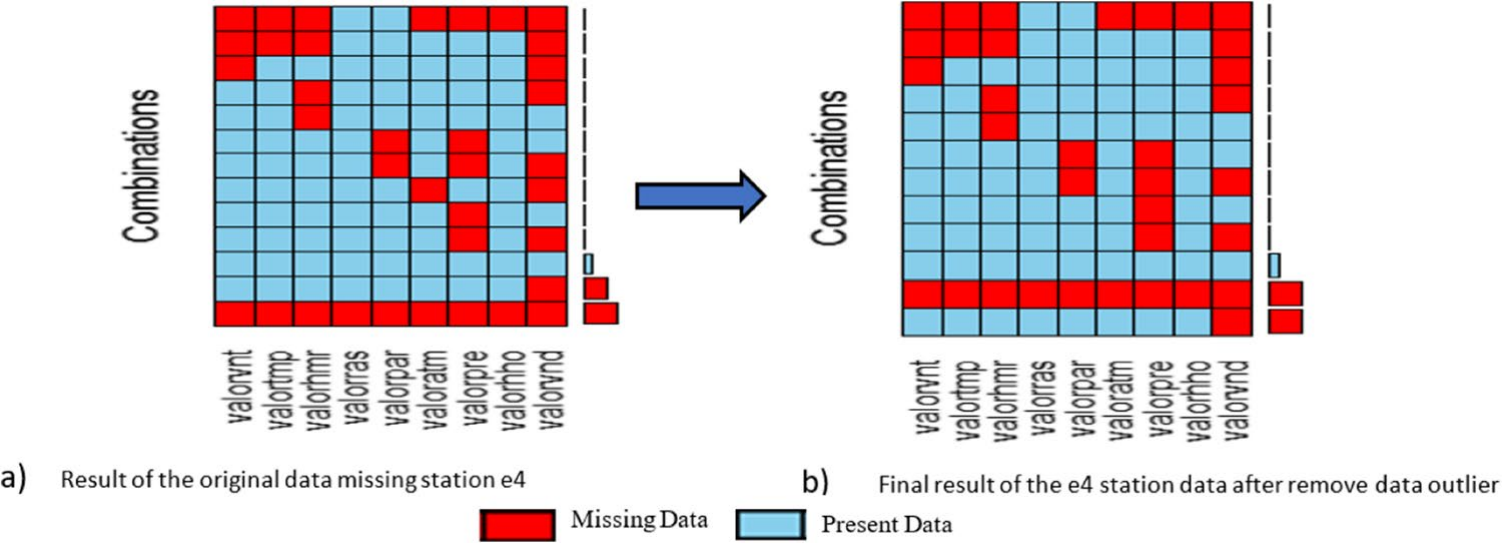
**a**, **b** Result of standardizing the data for the implementation of the algorithms to complete data on the 9 selected variables of the station E4

To standardize data from a total of 863.523 records between December 8, 2015, at 10:04 AM, now the Palmor station has a total of 863.823 records that go from December 08, 2015, at 10:47 AM to 30 July 2017 at 7:49 AM and have a percentage of missing data close to 41%. AM as of July 30, 2017, until 7:49 AM with a missing data of 41 percent, see Fig. 10.

**Fig. 11:**
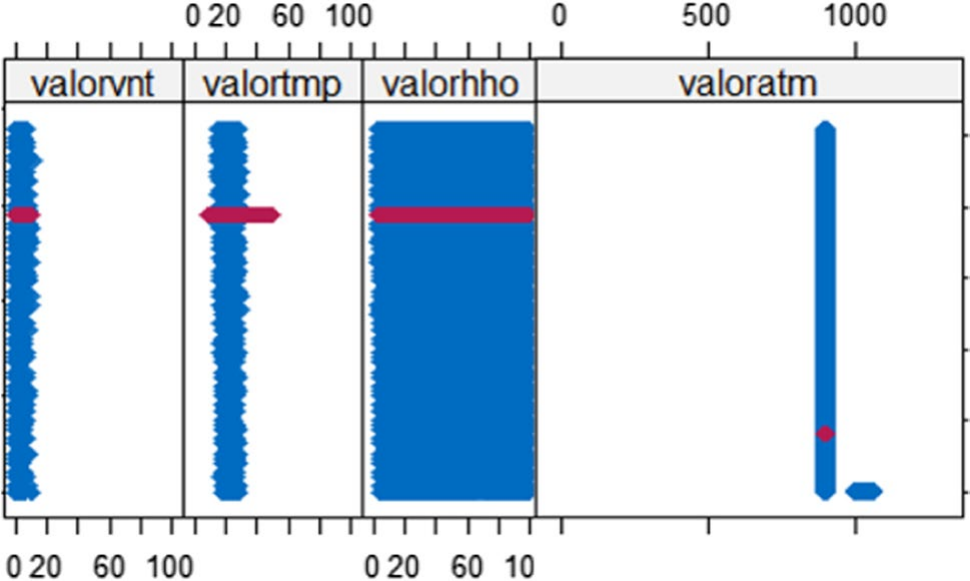
Result of the application of the mean function of the MICE package of R

The result of the mean function of the MICE package results in 4 variables, the behavior of completing the missing data in Fig. 11. The magenta points (imputed) coincide with the blue points (observed), which leads to conclude that they are “plausible values” and valid for the subsequent analysis in the prediction process. The result is acceptable for the mean function and makes the process suitable and capable of responding to complete data in a multivariable way by means.

**Fig. 12:**
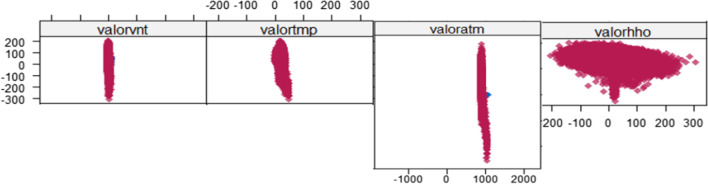
Result of the application of the norm.nob function, which is a linear regression with uncertainty

For the norm.nob method, which is linear regression without parameter uncertainty algorithm, its application and result are presented in Fig. 12. As a result of the analysis of the same 4 variables studied, it is observed that the imputed data coincide with the observed data, and a better expectation is fulfilled to complete the data, showing that it is a good algorithm to implement multivariable.

**Fig. 13:**
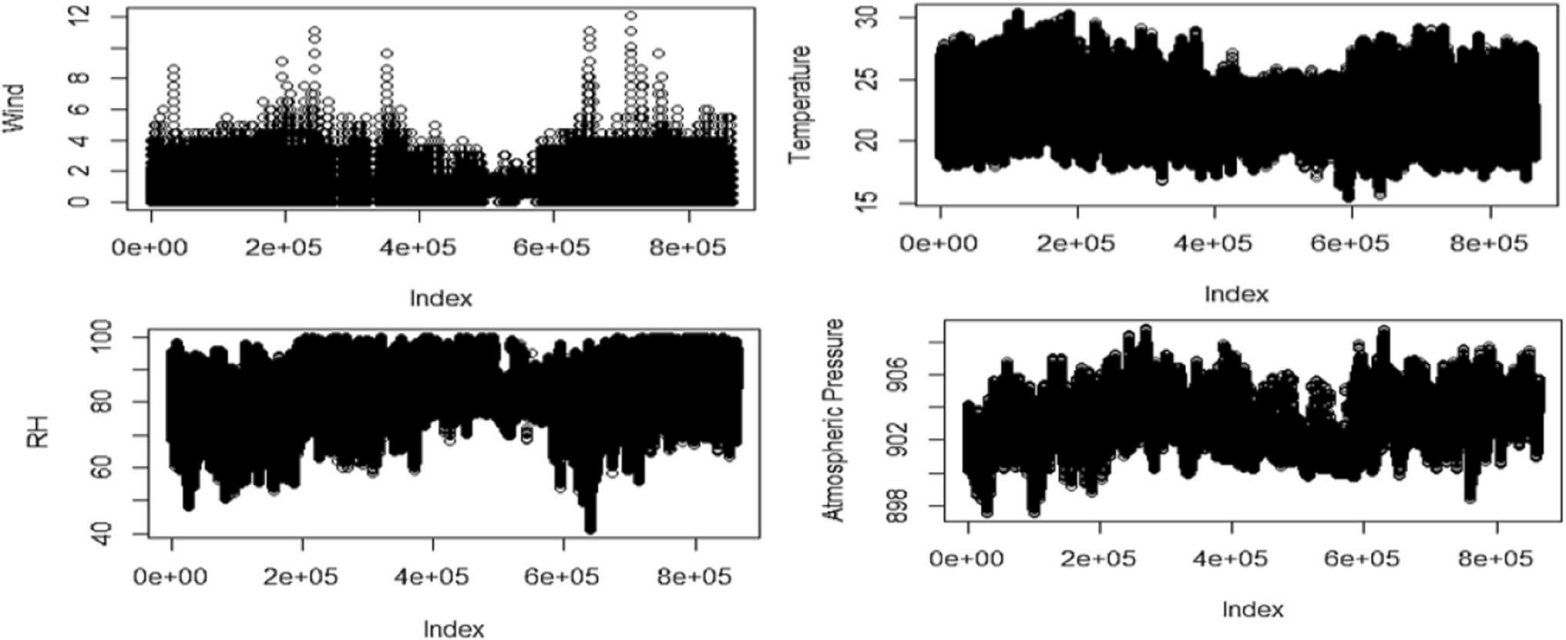
Result of the application of the DTF-IFFT algorithm developed in R

Finally, we have the result of the algorithm based on DTF-IFFT that is based on using the discrete Fourier series, and the result of this algorithm to complete data is presented in Figure 13, the same four variables of the 8 available from the station E4. It is appreciated that it completes the missing data in an efficient way and in its entirely fulfilling great performance, and the expectations of the result can be taken as very outstanding in the multivariable case. These three numerical models comply with their function to compile data to environmental variables that are supplied by meteorological stations.

Final analysis and results of the three algorithms to complete the data were achieved in each one, a solution of how to complete lost data, and it arrives that the DTF-IFFT algorithm complies with the best performance to complete the lost data due to different situation acquisition and transmission of data variables for each weather stations.

## Analysis and discussion

**Fig. 14:**
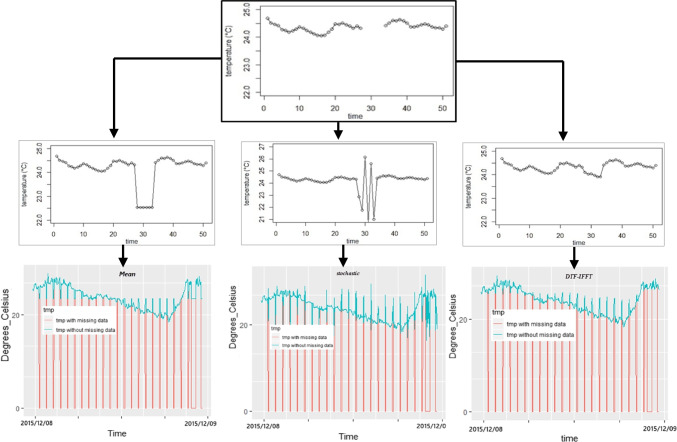
Result of the application of the DTF-IFFT algorithm developed in R

Due to the weakness of the data acquisition by the weather stations and their storage in the DB, the atypical data are presented: outliers and loss of information. The results of the three algorithms fulfill the expectations raised in the project, and these are presented in Fig. 14.

In Fig. 13, the result of the three algorithms to complete data is shown, and a part of the series is used with a data gap due to outlier or lost data. In the left part (second row), we have the result of the media technique, and a line was filled filling the gap but at a distance from the data of the original series. Second, the stochastic method (half) completes the data but its result varies, and finally, the DFT-IFFT (right) in the same vacuum is observed which completes harmoniously and smooths the missing data. From the above, we can observe the best performance of the DTF-IFFT and its result as the best method to complete a series of temporary data.

In the lower part of Fig. 14, the complete time series is shown, and in blue, it is shown how the data were filled for each method; it is observed that the most continuous and periodic is the DTF-IFFT in all its extension. This leads the completed data being more in agreement with the behavior of the temperature variable. In the case of DTF-IFFT, it was necessary to eliminate the initial data because they deteriorate the result, but when excluding data that do not have a periodicity, it was found that the algorithm improves in its performance; this did not occur in the mean and stochastic, the latter being more insensitive to this situation. The foregoing implies that using the DTF-IFFT is advisable to verify the periodicity and quantity of the data and that the amount of loss of information is not very large.

Based on the literature review and the results obtained, Schmitt et al. (2015) find that the mean, stochastic, and DTF-IFFT methods have an adequate performance, as they also find that they meet indicators such as root-mean-square error (RMSE) measures, a situation that is stronger for the DTF-IFFT method, leaving a greater degree of accuracy. Another example is by Caldera et al. (2016) who developed the comparison of seven methods of imputation and concludes that it is not possible to have a universal method because this depends on factors such as the amount of information and its quality, as well as the number of stations in the neighborhood for this purpose. Collect a good volume of data that allows to evaluate the different algorithms, concluding that it is not possible to determine a method, this situation is possible to be present because the stations have great variation of the information collected and the way it is willing each one, being an effect that should be evaluated in future works. Finally, Kim et al. (2019) show that in their research, a pre-processing of data improves the performance of the imputation methods situation that occurred in the case of DTF-IFFT delivering a better performance.

**Fig. 15:**
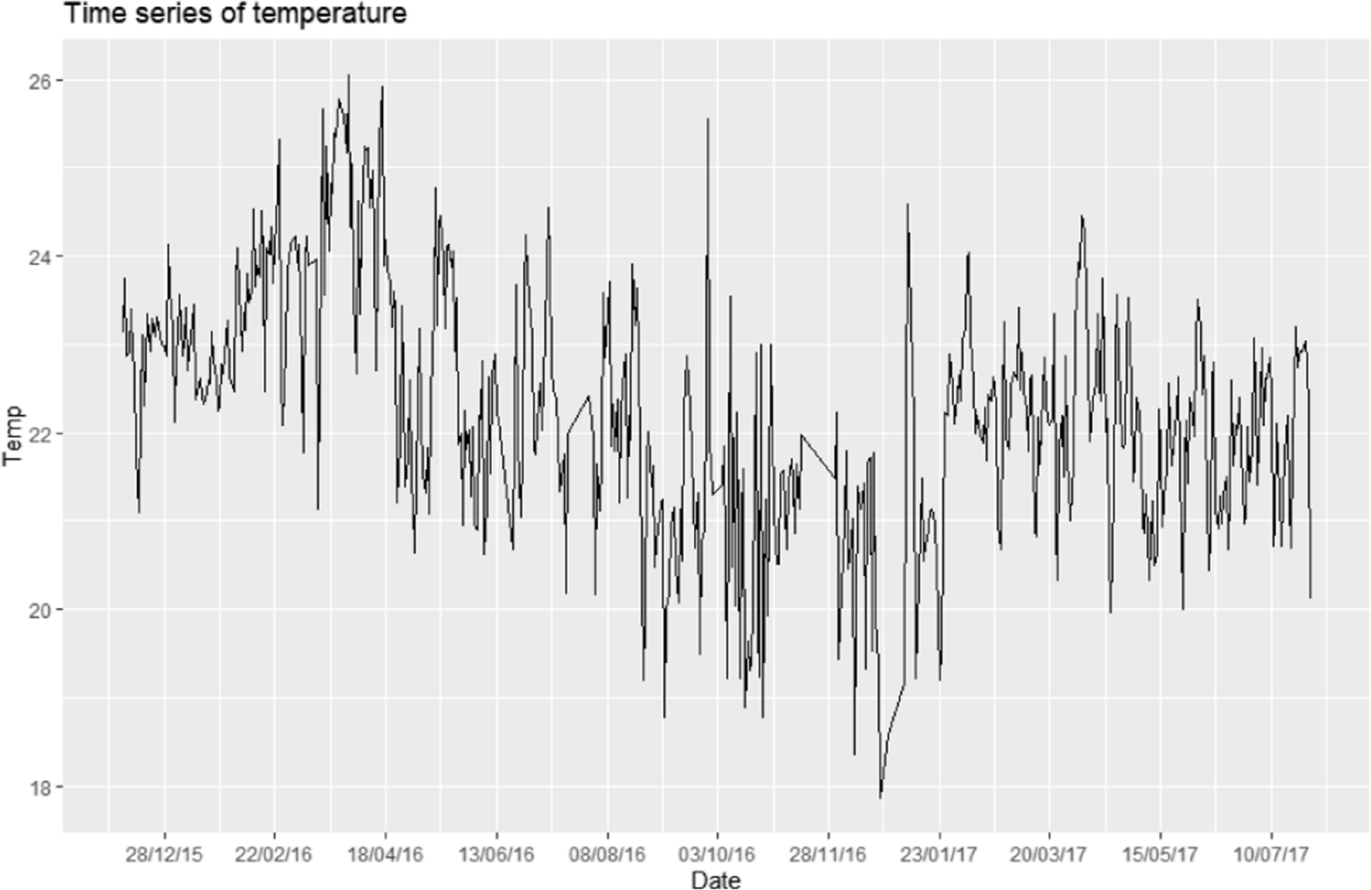
Results of the clean time series

To finish, the cleaned time series was reviewed with the complete data, and to establish its stationary behavior, for this, the procedure presented by Saleh et al. (2022) is followed, which describes a strategy to determine whether the time series is stationary. The results are the following:

First, the clean data of the series under analysis is plotted, showing the following result in Fig. 15.

Next, calculate the Dickey-Fuller test, and the result is as follows:

Dickey-Fuller=−4.5239, Lag order = 5, *p*-value < 2.2e−16

Based on the above results it can be observed that the *p*-value is less than 0.0001, indicating that in the Ho hypothesis, there is a unit trace present in the time series that can be rejected, suggesting the result that the time series under analysis is stationary.

El valor del Box-Cox.lamba es

[1] 1.999924

**Fig. 16:**
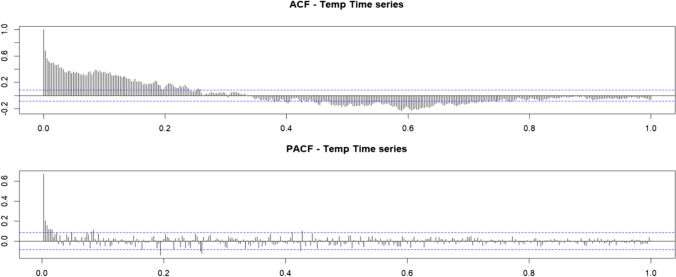
Results of the clean time series

The ACF and PACF results are plotted as shown in Fig. 16.

As a final part of verifying the data obtained from the Dickey-Fuller test and the Box-Cox, we proceed again to apply it, calculating the value of $\left(T\left({X}_t\right)=\sqrt{X_t}\right)$ and finally calculating diff1; the result is as follows:

Dickey-Fuller =−4.5351, Lag order = 5, *p*-value < 2.2e−16

lambda_2 <- BoxCox.lambda

> print(lambda_2)

[1] 1.999924

> serie_diff <- diff(serie_transf, differences = 1)

**Fig. 17:**
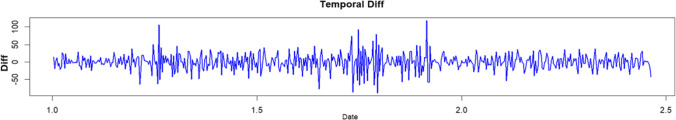
Results of the ADF test and time series plot of the first difference (diff1)

It is concluded that the *p*-value < 2.2e−16, concluding that the time series is stationary, and based on the ADF data, it is found that the *p*-values are below alpha 0.5, proving that the data under analysis are stationary, see Fig. 17.

## Conclusions

The result of this work is focused on the implementation of an outlier elimination methodology and complete data loss due to the acquisition of information through meteorological stations. We sought to study three methods with different numerical strategies and the DTF-IFFT method. It presents a good performance for the next prediction phase; this is based on the amount of information to be processed. It also has the result of the experience required to pre-process the information and establish transmission parameters with a constant latency to develop homogeneity of the data and thus have a sufficient amount of information. The result also contributed that the application of the DTF-IFFT method is more stable by its numerical base compared with the other two methods.

Monitoring using weather stations is essential for the development of flood forecasting tools; these are preliminary stages that must be detailed in order to choose the best model to complete data on meteorological variables. One of the difficulties is that the data arrive complete and that they are in the correct range for each variable which implies recourse to these numerical techniques. One of the results of the DFT-IFFT method is that better performance has to complete data and satisfy the multivariable process. The characteristics of the comparative model allow us to have an experimental base with DFT-IFFT to validate data from different stations and be part of the pre-process for the prediction of floods. The experimental process was performed on a developed DB that contained data from a period of time, the analysis of the three algorithms was done offline, and as a result, it is necessary to implement this strategy but online to verify the performance of the algorithms and in particular the DFT-IFFT. And this paper describes how models of outlier detection and lost data were implemented, as well as algorithms to complete a data set in Ciénaga Magdalena, Colombia. The future work is oriented to the online implementation for the flood forecasting process.

## Data Availability

Majority of the data were presented in this manuscript. Additional raw data may be made available upon request.
